# Validation and extension of the SUCCESS score for Fuchs dystrophy after cataract surgery

**DOI:** 10.1186/s40662-026-00501-4

**Published:** 2026-07-10

**Authors:** Francisco Arnalich-Montiel, Martha Stokking, Nerea Saenz-Madrazo, Jaime Etxebarria-Ecenarro, Pedro Arriola-Villalobos, Maria Gessa-Sorroche, Ana Martín-Ucero, Alfonso Muriel, Anabel Blasco-Moreno, Ana Vázquez-Fariñas, Verónica Gomez-Calleja, Francisco J. Muñoz-Negrete, David Mingo-Botín

**Affiliations:** 1 Cornea Unit, Department of Ophthalmology, Hospital Universitario Ramón y Cajal, Universidad de Alcalá, and Instituto Ramón y Cajal de Investigación Sanitaria (IRYCIS), Carretera de Colmenar Viejo km 9.1, 28034 Madrid, Spain; 2 University Center for Health Sciences – HM Hospitals (CUHMED), Camilo José Cela University, Madrid, Spain; 3 HM Hospitals Health Research Institute, Madrid, Spain; 4 Department of Ophthalmology, Hospital General Universitario Gregorio Marañón, Madrid, Spain; 5 Department of Ophthalmology, BioCruces Bizkaia Health Research Institute, Hospital Universitario de Cruces, Barakaldo, Bizkaia Spain; 6 Department of Ophthalmology, Hospital Clínico San Carlos and Instituto de Investigación Sanitaria San Carlos (IdISSC), Madrid, Spain; 7 Cornea, Cataract and Anterior Segment Unit, Hospital Universitario Virgen Macarena, Sevilla, Spain; 8 Ophthalmology Service, Hospital Universitario La Paz, Madrid, Spain; 9 Biostatistics Unit, Ramón y Cajal University Hospital, IRYCIS, and CIBERESP, Madrid, Spain; 10 Servei d’Estadística Aplicada, Universitat Autònoma de Barcelona, Barcelona, Spain; 11 Departament de Matemàtiques, Universitat Autònoma de Barcelona, Cerdanyola del Vallès, Spain

**Keywords:** Fuchs endothelial corneal dystrophy, Cataract surgery, Endothelial keratoplasty, Scheimpflug imaging, Risk prediction model

## Abstract

**Background:**

Accurate preoperative identification of patients with Fuchs endothelial corneal dystrophy (FECD) who are at risk of requiring endothelial keratoplasty (EK) after cataract surgery remains challenging. The SUbClinical Corneal Edema Scheimpflug Study (SUCCESS) score was previously developed to estimate this risk using Scheimpflug tomography. We aimed to prospectively and externally validate the SUCCESS score in an independent multicenter cohort and determine whether the incorporation of corneal densitometry improves predictive performance and clinical utility.

**Methods:**

In this prospective multicenter cohort study, 207 eyes from 177 patients with FECD grade ≥ 2 and visually significant cataract were enrolled across six tertiary hospitals in Spain (June 2020 to October 2024), with follow-up through May 2025. After predefined exclusions, 178 eyes (149 patients) were analyzed. The original SUCCESS score was used without recalibration. An extended model incorporated peak mean corneal densitometry within the central 3-mm zone. Time-to-event analyses were performed using Cox proportional hazards models. Discrimination, calibration, reclassification, and clinical utility were assessed using Harrell’s C-index, Brier score, net reclassification index (NRI), integrated discrimination improvement (IDI), and decision curve analysis.

**Results:**

Over a median follow-up of 37.0 months (interquartile range [IQR], 10.1–45.9), 38 eyes (21%) met the criteria for EK, with 82% of events occurring within 6 months after phacoemulsification. The original SUCCESS score demonstrated good discrimination (C-index = 0.80; 95% confidence interval [CI], 0.73–0.86) with slight underestimation of absolute risk (predicted 16.4% vs. observed 21.6%). Incorporation of corneal densitometry improved discrimination (C-index = 0.85; 95% CI, 0.79–0.90; ΔC = + 0.05; *P* = 0.0047), reduced prediction error (ΔBrier = − 0.019; *P* < 0.001), and enhanced risk reclassification (NRI = 0.39; 95% CI, 0.14–0.64; *P* = 0.002), including correct upward reclassification of 26% of eyes requiring EK. The extended model provided greater net benefit at relevant decision thresholds (25%–50%).

**Conclusions:**

In this multicenter external validation study, the SUCCESS score demonstrated a robust performance in predicting postoperative EK in FECD. The addition of corneal densitometry provided incremental improvements in discrimination and clinically meaningful risk stratification, particularly in intermediate-risk cases. Standardized Scheimpflug-based risk assessment may support individualized surgical planning and referral decisions.

**Supplementary Information:**

The online version contains supplementary material available at 10.1186/s40662-026-00501-4.

## Background

Fuchs endothelial corneal dystrophy (FECD) is the most common indication for endothelial keratoplasty (EK) in developed countries and frequently coexists with age-related cataract. Corneal transplantation after phacoemulsification has been reported to occur up to 68 times more frequently in eyes with corneal guttata than in the general population [1]. Cornea guttata, particularly its advanced form, most commonly reflects FECD. Despite this well-recognized risk, accurately identifying which eyes will tolerate cataract surgery alone and which will ultimately require EK remains a major clinical challenge.

Scheimpflug tomography has enabled earlier and more objective detection of subclinical corneal edema, an early manifestation of endothelial dysfunction, before the onset of clinically evident decompensation. Characteristic tomographic features, including the loss of regular isopachs, defined as the disruption of the normal smooth concentric pachymetric contour pattern, displacement of the thinnest point, and focal posterior corneal depression, have been shown to predict disease progression and postoperative corneal failure [2].

Building on these observations, we previously developed a multivariable prediction model integrating central corneal thickness (CCT) with Scheimpflug-derived morphological features of subclinical corneal edema to estimate the risk of requiring EK after uncomplicated cataract surgery for FECD [3]. This model was subsequently termed the SUbClinical Corneal Edema Scheimpflug Study (SUCCESS) score to facilitate communication and clinical implementation. This score provides an objective and reproducible method for estimating the likelihood of requiring EK after cataract surgery, supporting more informed surgical planning and patient counseling. However, external validation in independent multicenter cohorts is essential before its widespread clinical adoption. Notably, such a prediction model does not determine whether EK will be performed. Rather, it estimates the preoperative likelihood that the postoperative corneal status will lead to a clinical recommendation for EK. The actual decision to proceed with keratoplasty remains individualized and may be influenced by postoperative visual function, patient symptoms, fellow-eye status, systemic comorbidities, patient preference, and intraoperative factors that cannot be fully captured preoperatively.

Moreover, although Scheimpflug tomography captures the morphologic manifestations of edema, early FECD may also be characterized by diffuse optical changes not fully reflected by pachymetric or elevation-based parameters. Corneal densitometry, derived from the same imaging platform, quantifies backscattered light and may capture complementary aspects of corneal compromise [4, 5].

Accordingly, the present study aimed to externally validate the SUCCESS score in a prospective multicenter cohort and assess whether incorporating corneal densitometry improves its predictive performance and clinical utility for individualized surgical decision-making in FECD-associated cataract surgery.

## Methods

### Study design and participants

The SUCCESS is a prospective, multicenter national diagnostic study conducted across six tertiary referral centers in Spain: Hospital Universitario Ramón y Cajal, Hospital General Universitario Gregorio Marañón, Hospital Universitario de Cruces, Hospital Clínico San Carlos, Hospital Universitario Virgen Macarena, and Hospital Universitario La Paz. The study aims to assist ophthalmologists in assessing and communicating the risk of postoperative corneal decompensation after cataract surgery in patients with FECD.

Between June 2020 and October 2024, adult patients diagnosed with FECD grade ≥ 2 on the modified Krachmer scale [6] (> 12 scattered guttae) and visually significant cataract were consecutively recruited and scheduled for cataract surgery. All patients included in the present analysis were newly enrolled during this period and previously published model coefficients were applied without recalibration, fulfilling the accepted criteria for prospective temporal and multicenter external validations.

Follow-up continued until May 2025, including the systematic reassessment of patients not requiring EK. Patients with microcystic or bullous epithelial edema were scheduled for combined phacoemulsification and EK and were therefore excluded. Additional exclusion criteria included prior ocular trauma or surgery, ocular inflammation or infection, concurrent ocular disease other than cataract or FECD that could significantly affect visual function (e.g., advanced glaucoma, macular degeneration, or other retinal pathology), intellectual disability or dementia, inability to complete follow-up, or intraoperative complications during phacoemulsification. If both eyes were operated on during the study period, both were included.

This study adhered to the Declaration of Helsinki and was approved by the institutional review boards of all participating centers (ID 062-20). Written informed consent was obtained from all the participants. The study was registered at ClinicalTrials.gov (NCT07265388) and conducted in accordance with the transparent reporting of a multivariable prediction model for individual prognosis or diagnosis (TRIPOD) statement [7] for transparent reporting of prediction model validation. A summary of TRIPOD compliance is shown in Supplementary Table S1.

### Clinical assessment

All patients underwent standardized preoperative evaluation 1–3 months before surgery, including medical and ophthalmic history, slit-lamp examination, FECD staging according to the modified Krachmer scale [6], and cataract grading using the Barcelona Classification [8]. Cataracts were considered visually significant when lens opacity was judged by the treating surgeon to contribute meaningfully to the patient's visual symptoms or functional limitation because visual acuity in FECD may be affected by both cataracts and corneal dysfunction; no isolated visual acuity threshold was used as the sole criterion for cataract significance.

Scheimpflug tomography (Pentacam HR; Oculus, Wetzlar, Germany; software version 1.20r112) was performed between 9:00 a.m. and 12:00 p.m., at least 2 h after awakening. Endothelial cell density was assessed by specular microscopy using the non-contact instrument available at each participating center. Because this parameter was not included as a predictor in either the base or extended SUCCESS models, inter-device variability did not affect score calculation. Macular integrity was confirmed by optical coherence tomography.

Clinical and imaging data were entered into a secure REDCap web-based registry.

Postoperative visits were routinely scheduled for 6–10 weeks. Additional visits were performed at the surgeon’s discretion when a longer observation period was considered clinically appropriate before establishing a surgical recommendation.

Cataract surgery was performed by 10 board-certified anterior-segment or corneal surgeons with a median independent surgical experience of 15 years (range, 10–25 years). All surgeons were members of the corneal unit at their respective institutions and routinely performed phacoemulsification in eyes with FECD. Surgical duration and surgeon experience were recorded and explored as potential predictors of postoperative EK indication.

### Surgical decision criteria

The primary outcome was indication for EK after cataract surgery.

The decision to recommend EK was based on predefined functional and clinical criteria:

1. Postoperative best spectacle-corrected visual acuity ≥ 0.30 logMAR; and/or
2. Vision-impairing symptoms attributable to corneal disease (e.g., morning blurring, halos, starbursts, or difficulty focusing) interfering with daily activities.

All patients completed the VF-14 visual function index before cataract surgery and again before any EK decision to quantify vision-related functional limitations and support the clinical judgment underlying the surgical recommendation [9, 10]. The VF-14 scores were not used as independent predictors in the statistical models.

Each case was independently reviewed by a second ophthalmologist (FAM) blinded to the initial recommendation, and discrepancies were resolved through discussion with a third corneal specialist (DMB) when necessary. Patients retained the right to decline EK after counseling, and reasons for refusal were recorded.

Although the first postoperative visit occurred at 6–10 weeks, the indication for EK was treated as a time-to-event outcome and could be established at any point during the follow-up. For all participants, we recorded the time from cataract surgery to the indication for EK or last follow-up (censoring).

### Calculation of the SUCCESS score

The previously published prediction model [3] was applied as originally described. Briefly, the score combines the number of Scheimpflug-derived tomographic features consistent with subclinical corneal edema (as defined by Sun et al.) with the categorized CCT, yielding a total score ranging from 0 to 8 points. The model, originally derived from Cox proportional hazards regression and subsequently translated into a point-based system for clinical use, was directly applied to this cohort without recalibration to assess its reproducibility and generalizability.

This prediction model was subsequently termed the SUCCESS score to facilitate communication and clinical implementation.

### Extension of the SUCCESS score

We investigated whether the addition of routinely available imaging parameters could enhance the prediction of postoperative EK. Corneal densitometry was prespecified as a candidate variable because increased backscatter may precede clinically evident edema and capture early tissue alterations that are not fully reflected in pachymetric or elevation-based indices [4, 11].

All densitometric parameters were obtained using the same Pentacam Scheimpflug system used for tomographic and pachymetric measurements to ensure methodological consistency across the centers. The measurements were exported directly from the device without external normalization, reflecting real-world clinical practice.

Specifically, the anterior, central, posterior, and total densitometry values within the central 2-mm zone were recorded. The peak mean corneal densitometry value was defined as the highest value from the mean densitometry map within the central 3-mm apex-centered zone, a parameter readily available in the standard Pentacam output. A representative Pentacam HR report showing where this value can be identified is shown in Supplementary Figure S1. This metric was selected because it demonstrated the strongest discriminative performance among the evaluated densitometric parameters, while remaining simple to obtain in routine clinical practice.

After evaluating several quantitative parameters individually, including layer-specific densitometry values, relative pachymetry, and anterior chamber depth, peak mean corneal densitometry within the central 3-mm zone showed the best overall discriminative performance (Supplementary Table S2). Using receiver operating characteristic (ROC) curve analysis, the optimal threshold for predicting EK indication was identified at 21.2 gray-scale units (GSU) as the value that jointly maximized sensitivity and specificity (Supplementary Figure S2).

The regression coefficient (β = 1.94) for the dichotomized densitometry variable corresponded approximately to six units on the original score scale (β ≈ 0.33 per point). However, assigning six additional points resulted in excessive dispersion of the score categories. Therefore, the densitometry variable was assigned four additional points for values ≥ 21.2 GSU to preserve proportional weighting while maintaining interpretability and clinical usability. This web-based calculator applies the continuous Cox regression equation rather than a simplified point-based scoring system. The model performance was evaluated using discrimination, calibration, and reclassification metrics in accordance with TRIPOD recommendations.

### Study objectives

The primary objective of this study was to externally validate the predictive performance of the SUCCESS score in identifying patients at risk of requiring EK after cataract surgery for FECD.

The secondary objective was to determine whether the inclusion of corneal densitometry improves the predictive accuracy, calibration, reclassification, and clinical utility of the extended SUCCESS model.

### Statistical analysis

This study was designed as a prospective external validation of an existing predictive model. Accordingly, the sample size rationale was based on the expected precision of the model performance estimates rather than a treatment-effect hypothesis test. Assuming an EK indication rate of approximately 20%, recruitment of approximately 150–180 eyes was expected to yield at least 30–35 events, allowing estimation of discrimination, calibration, and clinical utility with acceptable precision for external validation.

Continuous variables were summarized as medians (interquartile range [IQR]) and categorical variables as frequencies (percentages). Group differences were evaluated using the t test or Wilcoxon rank-sum test for continuous variables and the χ^2^ test for categorical variables.

Time-to-event analyses were performed using Cox proportional hazards models. Model discrimination was quantified using Harrell’s C-statistic (C-index), and calibration was assessed using calibration-in-the-large, calibration slopes, and graphical calibration plots at 12 months. The Brier score was calculated as an overall measure of the model accuracy.

To compare the original and extended models, we evaluated the changes in the C-index and Brier scores, and calculated the integrated discrimination improvement (IDI) and net reclassification improvement (NRI) at 12 months. Decision curve analysis (DCA) was used to assess clinical utility across clinically relevant threshold probabilities.

Because some patients contributed both eyes, sensitivity analyses were conducted using Cox proportional hazards models with robust variance estimation clustered at the patient level (base model: hazard ratio [HR] = 1.635; 95% confidence interval [CI], 1.395–2.001; extended model: HR = 1.515; 95% CI, 1.362–1.720) and by restricting the analysis to the first operated eye per patient (base model: HR = 1.580; 95% CI, 1.333–1.873; extended model: HR = 1.428; 95% CI, 1.271–1.605). In both cases, the HRs and CIs were consistent with those of the primary analysis, indicating a minimal impact of within-patient correlation. Therefore, the primary results are presented using the standard Cox model, including all eyes.

All tests were two-sided, and statistical significance was set at *P* < 0.05. Statistical analyses were performed using the R software (version 4.5.2; R Foundation for Statistical Computing, Vienna, Austria).

## Results

### Patient characteristics and follow-up

**Fig. 1:**
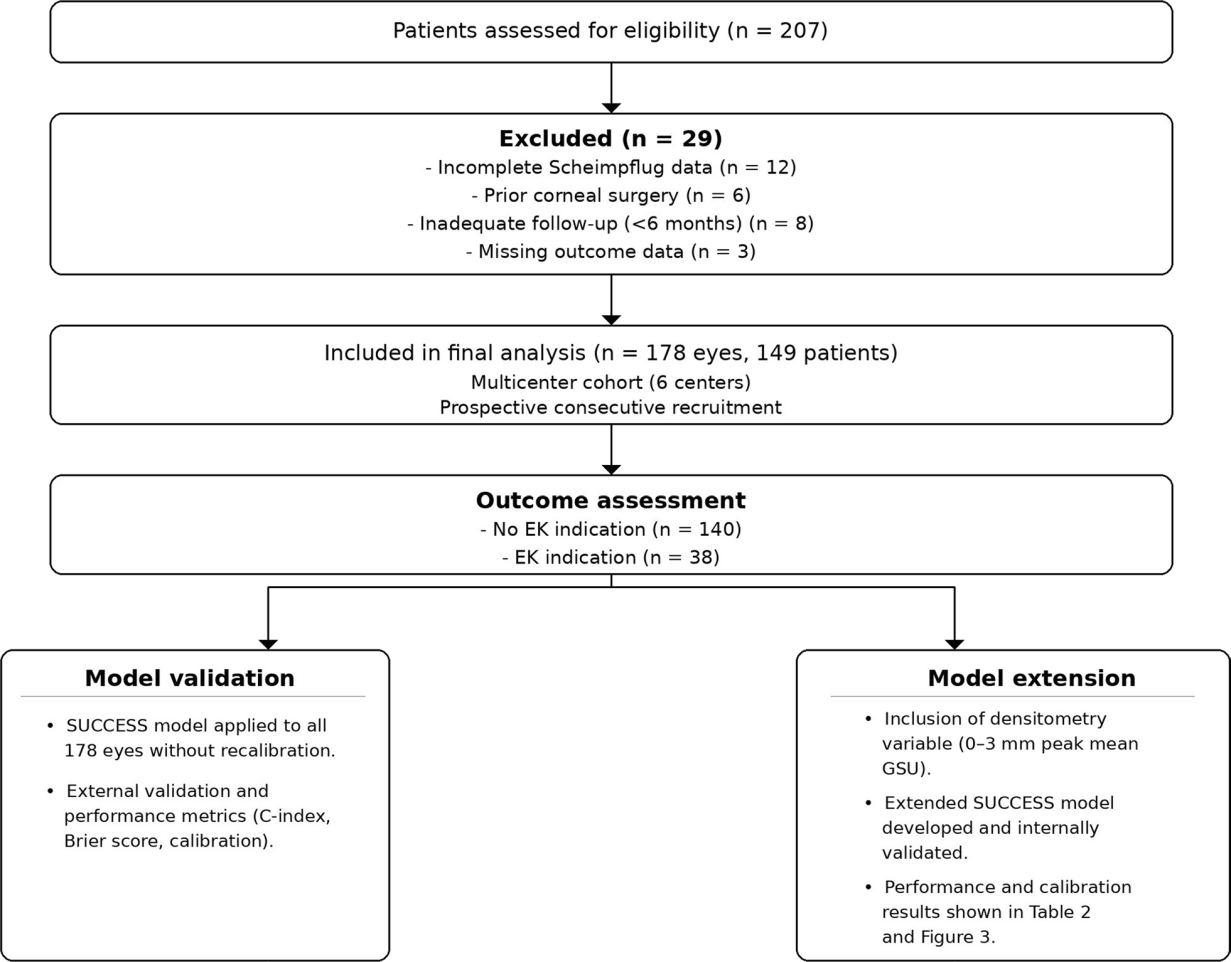
Flowchart of patient selection and analysis. Of 207 eyes assessed for eligibility, 29 were excluded due to incomplete Scheimpflug data (n = 12), prior corneal surgery (n = 6), inadequate follow-up (< 6 months; n = 8), or missing outcome data (n = 3). A total of 178 eyes from 149 patients were included across six tertiary centers with prospective consecutive recruitment. The original SUbClinical Corneal Edema Scheimpflug Study (SUCCESS) model was externally validated in this cohort, and an extended version incorporating corneal densitometry (0–3 mm maximum mean gray-scale units) was developed and internally validated

Between June 2020 and October 2024, 207 eyes from 177 patients were enrolled in the study. Twenty-nine eyes (14%) were excluded because of incomplete tomographic data, insufficient follow-up, or protocol deviations (e.g., previous laser-assisted in situ keratomileusis, shagreen degeneration affecting densitometry, or other ocular comorbidities). Three additional patients were excluded because of postoperative complications that compromised visual outcomes. The study flow diagram summarizing patient inclusion, exclusion, and final analysis populations is presented in Fig. 1.

The final cohort comprised 178 eyes from 149 patients (86% of those recruited). During follow-up, 38 eyes (21%) met the predefined indication criteria for EK based on functional and morphological assessment, although 31 eyes (17%) ultimately underwent the procedure. In the remaining seven eyes, surgery was deferred or declined because of satisfactory binocular visual function (n = 2), systemic comorbidities (n = 3), or personal preference (n = 2). None of the patients underwent EK or requested it in the absence of clinical indications.

The median follow-up time after cataract surgery was 37.0 months (IQR, 10.1–45.9 months), reflecting extended administrative reassessment through May 2025. Among eyes meeting criteria for EK, the median time from cataract surgery to EK indication was 2.74 months (IQR, 1.81–4.96 months). Notably, 81.6% of EK indications occurred within the first 6 months and 92.1% within the first 12 months after surgery.

Recruitment varied across the six centers (6%–31% per site), but the proportion of EK indications did not differ significantly among hospitals (*P* > 0.05), suggesting consistency of outcomes across institutions.

Eyes from patients contributing only one eye to the study had a higher rate of EK indication (26%) than eyes from bilateral cases (12%; *P* = 0.04). This likely reflects the clinical sequencing of surgery between fellow eyes, rather than biological independence.

The median age of participants was 71 years (IQR, 67–77 years), and 67% were women. The baseline characteristics of the study cohort are summarized in Table 1. Compared with eyes that did not require EK, those meeting the criteria for EK were generally younger and exhibited more advanced markers of endothelial dysfunction, including greater CCT, higher peak mean corneal densitometry values, and more advanced FECD stage. These eyes also demonstrated a greater number of tomographic edema features and a higher proportion of non-identifiable endothelial cells.

**Table 1:** Baseline characteristics of the study cohort

Characteristic	All FECD patients (n = 178)	No EK (n = 140)	EK required (n = 38)	*P* value
Age (years), median (IQR)	71 (66–78)	72.8 (67.7–77.8)	68.4 (64.0–71.0)	0.004
Female, No. (%)	117 (66.0)	95 (68.0)	22 (58.0)	0.25
Diabetes, No. (%)	20 (11.2)	14 (10.0)	6 (16.0)	0.33
FECD grade, No. (%)				< 0.001
1	15 (8.4)	15 (10.7)	0 (0)	
2	32 (18.0)	31 (22.1)	1 (2.6)	
3	35 (19.7)	31 (22.1)	4 (10.5)	
4	42 (23.6)	37 (26.4)	5 (13.2)	
5	34 (19.1)	19 (13.6)	15 (39.5)	
6	20 (11.2)	7 (5.0)	13 (34.2)	
Cataract grade (BCN scale), median (IQR)				
Nuclear	4 (3–5)	4.5 (3–5)	4 (2–5)	0.016
Cortical	0 (0–2)	0 (0–1)	1 (0–2)	0.24
Posterior	0 (0–0)	0 (0–0)	0 (0–0)	0.43
Preoperative BSCVA (logMAR), median (IQR)	0.50 (0.40–0.63)	0.50 (0.40–0.63)	0.40 (0.32–0.50)	0.055
Central corneal thickness (μm), median (IQR)	574 (551–608)	565 (547–596)	614 (579–653)	< 0.001
0–3 mm peak mean densitometry (GSU), median (IQR)	19.3 (16.9–22.2)	18.4 (16.1–20.6)	24.7 (21.6–27.9)	< 0.001
Relative pachymetry ratio, median (IQR)	3.2 (1.7–6.0)	3.4 (1.4–5.0)	8.1 (4.1–11.0)	< 0.001
Identifiable endothelial cell count, No. (%)	84 (47.2)	52 (37.1)	32 (84.2)	< 0.001

*BSCVA* = best spectacle-corrected visual acuity; *EK* = endothelial keratoplasty; *FECD* = Fuchs endothelial corneal dystrophy; *GSU* = gray-scale unit; *IQR* = interquartile range

Data are presented as median (IQR) unless otherwise indicated

Additional exploratory imaging parameters not included in the predictive models, such as layer-specific densitometry and the relative pachymetry ratio, are presented in Supplementary Table S3. In the univariable exploratory analyses, nuclear cataract grade, surgical time, and surgeon experience were not significantly associated with EK indications (all *P* > 0.10).

### Performance of the base model (SUCCESS score)

The original SUCCESS score demonstrated good discrimination for predicting the risk of requiring EK after cataract surgery, with a Harrell’s C statistic of 0.80 (95% CI, 0.73–0.86).

At 12 months, the mean predicted risk was 16.4%, compared with an observed event rate of 21.6%, yielding a calibration-in-the-large difference of − 5.2%, indicating a modest underestimation of absolute risk. The calibration intercept was − 3.82, and the calibration slope was 0.99 (standard error, 0.17), supporting appropriate model calibration and minimal evidence of overfitting in this external cohort.

The Brier score at 12 months was 0.118 (95% CI, 0.086–0.148), indicating good overall accuracy. The model performance remained stable over time, with Brier scores of 0.122, 0.118, and 0.125 at 6, 12, and 24 months, respectively.

**Fig. 2:**
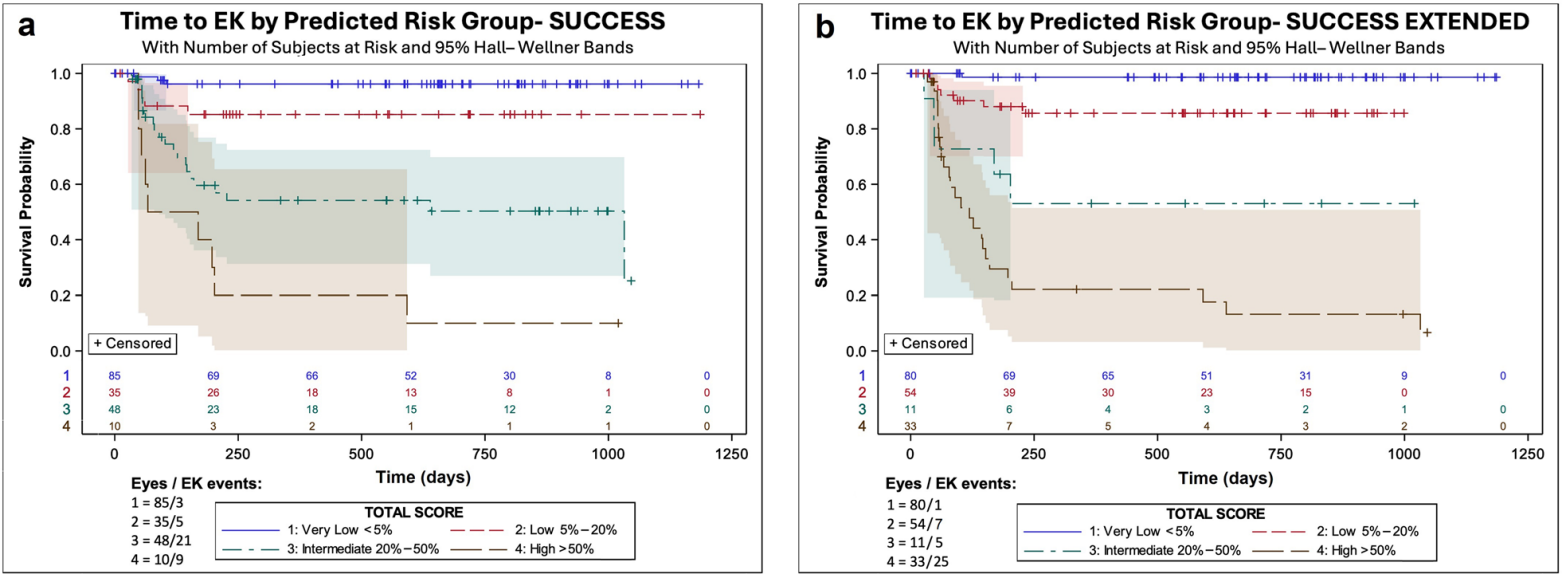
Kaplan–Meier curves showing time to endothelial keratoplasty (EK) indication by predicted-risk group according to the original SUbClinical Corneal Edema Scheimpflug Study (SUCCESS) model (**a**) and the extended SUCCESS model incorporating corneal densitometry (**b**). Shaded areas represent 95% Hall–Wellner confidence bands, and numbers at risk are displayed below each panel. Risk groups were categorized a priori as very low risk (< 5%), low risk (5%–20%), intermediate risk (20%–50%), and high risk (> 50%) predicted probability of EK indication. As shown in panel (**a**), the total number of eyes/EK indications in each risk group was 85/3, 35/5, 48/21, and 10/9 for the very low-, low-, intermediate-, and high-risk groups, respectively. For panel (**b**), the corresponding numbers were 80/1, 54/7, 11/5, and 33/25. These categories represent clinically interpretable risk strata and were not intended to contain an equal number of eyes. Separation between the strata was observed in both models (log-rank *P* < 0.001). The numbers of eyes and EK events within each risk category are displayed below each panel. The extended model reclassified a substantial proportion of eyes into different risk categories compared to the base model, as reflected in the redistribution of events across strata

Kaplan–Meier curves stratified by the four predefined risk categories (very low < 5%, low 5%–20%, intermediate 20%–50%, and high > 50%) demonstrated progressive separation over time (log-rank *P* < 0.001) (Fig. 2a). Numbers at risk and 95% confidence bands are displayed. The time axis represents the number of months from cataract surgery to EK indication or censoring during the most recent clinical evaluation. Proportional hazard assumptions were assessed using Schoenfeld residuals and showed no statistically significant violations.

**Fig. 3:**
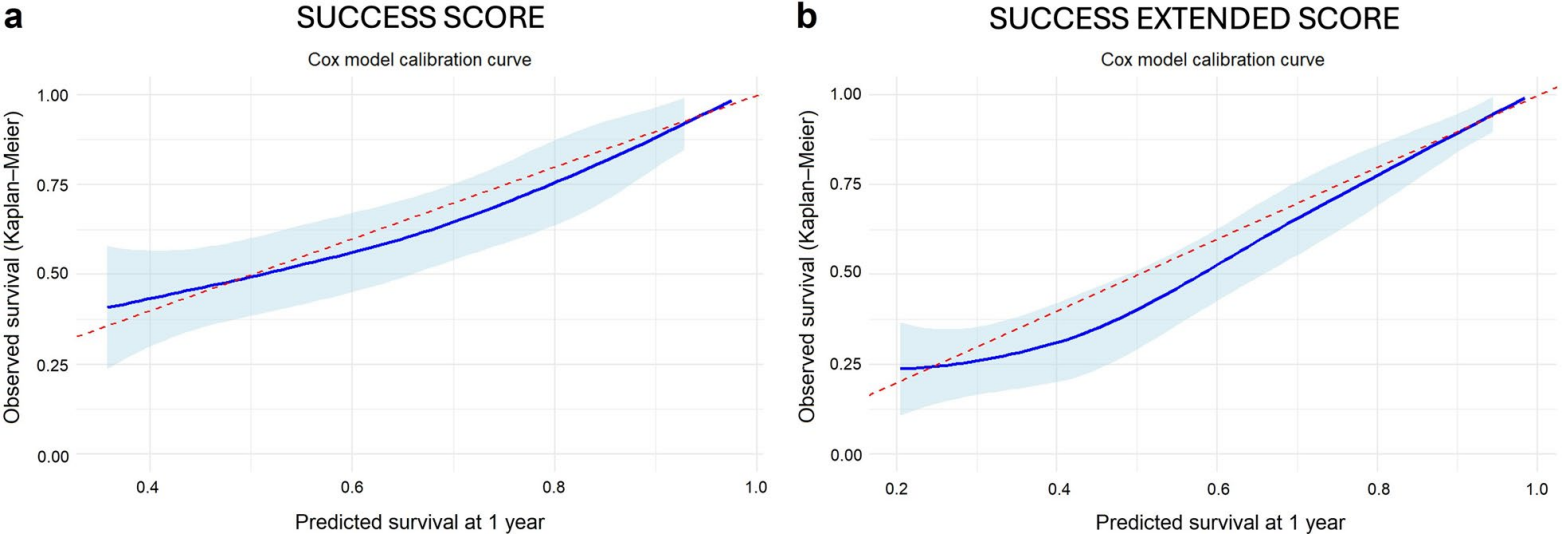
Calibration plots for the (**a**) original SUbClinical Corneal Edema Scheimpflug Study (SUCCESS) model and (**b**) the extended SUCCESS model including corneal densitometry. The x-axis represents predicted endothelial keratoplasty (EK)-free survival at 1 year, and the y-axis represents observed survival derived from Kaplan–Meier estimates. The dashed red line indicates perfect calibration, and the solid blue line represents model performance with 95% confidence bands. The extended model demonstrates closer agreement between predicted and observed probabilities, reflecting improved calibration

The calibration plot (Fig. 3a) showed a close agreement between the predicted and observed probabilities of not requiring EK after cataract surgery, with only mild underestimation in the intermediate probability range.

Overall, the base model demonstrated strong discrimination, appropriate calibration, and temporal stability in this multicenter cohort. To determine whether predictive performance and clinical classification could be further enhanced, we evaluated the incremental value of incorporating corneal densitometry parameters.

### Extended model incorporating corneal densitometry

To evaluate whether the predictive performance could be enhanced, the peak mean corneal densitometry value within the central 3-mm zone, obtained from the same Pentacam Scheimpflug system used for tomographic analysis, was assessed as an additional candidate variable. Because the relationship between densitometry and the risk of requiring EK was nonlinear, quantitative predictors were examined using ROC curve analysis to determine the optimal cutoff values by jointly maximizing sensitivity and specificity. For peak mean corneal densitometry within the central 3-mm zone, the optimal threshold was 21.2 GSU, which demonstrated the best discriminative performance among the evaluated densitometric parameters, and was therefore selected for model extension (Supplementary Figure S2).

When the dichotomized densitometry variable was added to the base model, model discrimination improved from C = 0.80 (95% CI, 0.73–0.86) to C = 0.85 (95% CI, 0.79–0.90) (ΔC = + 0.05; 95% CI, 0.02–0.09; *P* = 0.0047). Global accuracy also improved, with a mean Brier score reduction of − 0.019 ± 0.005 (*P* < 0.001) (Table 2). The temporal evolution of the Brier score demonstrated the consistent superiority of the extended model over time (Supplementary Figure S3).

**Table 2:** Performance comparison between the original and extended SUCCESS models

Metric	SUCCESS original	SUCCESS + densitometry	ΔSE	*P* value
Harrell’s C (95% CI)	0.80 (0.73–0.86)	0.85 (0.79–0.90)	+ 0.05 (± 0.019)	0.0047
Brier score	0.118	0.099	− 0.019 (± 0.005)	< 0.0001
Calibration slope	0.99	1.00	+ 0.01	–
Calibration intercept	− 3.82	− 2.50	+ 1.32	–
NRI (95% CI)	–	0.39 (0.22–0.56)	–	< 0.001
IDI	–	0.14	–	< 0.001

*CI* = confidence interval; *IDI* = integrated discrimination improvement; *NRI* = net reclassification index; *SE* = standard error; *SUCCESS* = SUbClinical Corneal Edema Scheimpflug Study

Harrell’s C and Brier scores represented the overall model discrimination and calibration, respectively. Brier score differences and *P* values were calculated using 2000 bootstrap resamples. Lower values indicate better calibration

Kaplan–Meier curves stratified by predicted risk categories derived from the extended model (Fig. 2b) showed improved risk stratification across predefined groups compared to the base model (Fig. 2a) (log-rank *P* < 0.001 for both models). Predicted probabilities were categorized into four clinically interpretable groups: very low (< 5%), low (5%–20%), intermediate (20%–50%), and high (> 50%) predicted risk. Numbers at risk and 95% confidence bands are displayed.

Calibration metrics also improved. The calibration intercept changed from − 3.82 in the base model to − 2.50 in the extended model, and the calibration-in-the-large difference narrowed from − 5.2% to − 2.2%, indicating reduced underestimation of risk. The calibration slope remained stable (1.00; standard error = 0.14), supporting the preservation of model stability. Calibration plots at 12 months (Fig. 3b) demonstrated a close agreement between the predicted and observed probabilities, with only minor deviations in the intermediate-risk range.

Reclassification analyses supported the incremental value of densitometry. At 12 months, the NRI was 0.39 (95% CI, 0.14–0.64; *P* = 0.002). This improvement was driven by correct upward reclassification in 26% of eyes that ultimately required EK and correct downward reclassification in 13% of eyes that did not require EK. The IDI was 0.14 (95% CI, 0.04–0.23; *P* = 0.014), reflecting greater separation of predicted risks between event and non-event groups.

The simplified point-based version of the extended score demonstrated calibration performance comparable to that of the continuous Cox model, indicating that score simplification does not materially compromise predictive accuracy.

Overall, the incorporation of corneal densitometry provided incremental improvements in discrimination, calibration, and reclassification, supporting its potential value for refining risk estimation in patients with FECD undergoing cataract surgery.

### Clinical utility

DCA (Table 3) demonstrated that both the base and extended models provided positive net benefit across a broad range of clinically relevant decision thresholds (10%–80%), outperforming the default strategies of “treat-all” (assuming all patients are at high risk of requiring EK) and “treat-none” (assuming none are at high risk).

**Table 3:** Clinical decision thresholds and net benefit analysis

Threshold probability (%)	SUCCESS original (NB)	SUCCESS + densitometry (NB)	ΔNB (95% CI)	Interpretation
10	0.161	0.171	+ 0.010 (− 0.010 to 0.033)	Not significant
15	0.143	0.152	+ 0.008 (− 0.014 to 0.027)	Not significant
20	0.129	0.147	+ 0.018 (− 0.006 to 0.040)	Borderline (+ ≈ one patient per 100)
25	0.109	0.143	+ 0.034 (0.003 to 0.061)	Significant, in favor of extended model (+ ≈ three patients per 100)
30	0.102	0.136	+ 0.034 (0.001 to 0.065)	Significant, in favor of extended model (+ ≈ three patients per 100)
40	0.058	0.111	+ 0.053 (0.004 to 0.107)	Significant, in favor of extended model (+ ≈ five patients per 100)
50	0.039	0.096	+ 0.057 (0.006 to 0.113)	Significant, in favor of extended model (+ ≈ six patients per 100)
60	0.042	0.073	+ 0.031 (− 0.031 to 0.099)	Not significant
75	0.000	0.023	+ 0.023 (− 0.059 to 0.090)	Not significant
80	0.000	0.034	+ 0.034 (0.008 to 0.068)	Significant, in favor of extended model (+ ≈ three patients per 100)

*CI* = confidence interval; *NB* = net benefit; *ΔNB* = difference in net benefit between the extended and original SUCCESS models; *SUCCESS* = SUbClinical Corneal Edema Scheimpflug Study

Net benefit quantifies the clinical utility by balancing true- and false-positive decisions across risk thresholds. The extended model with corneal densitometry (0–3 mm maximum mean GSU) provided meaningful improvements at thresholds of 25%–50%, corresponding to ≈ 3–6 additional correctly classified patients per 100 evaluated compared with the original SUCCESS model

The extended model showed consistently greater net benefit between threshold probabilities of 25%–50%, a range that typically reflects the highest degree of clinical uncertainty when planning cataract surgery in patients with FECD. Within this range, differences between models were statistically significant, with the largest improvement observed at the 50% threshold (ΔNB = 0.057; 95% CI, 0.006–0.113). This corresponds to approximately six additional patients per 100 being more appropriately classified with respect to their risk of requiring EK.

These findings align with the observed reclassification results, in which approximately one in four eyes that ultimately required EK were correctly shifted to a higher-risk category, and approximately one in eight eyes that did not require EK were shifted to a lower-risk category. Such refinements are particularly relevant for patients near clinical decision thresholds, where even modest improvements in risk estimation may influence referral pathways, perioperative planning, and assurance that patients at higher risk are managed in centers capable of providing EK if needed. Conversely, the absence of additional net benefit at higher thresholds (60%–75%) likely reflects that, at these levels of predicted risk, most patients are already correctly identified as high risk by the base model, and management decisions are typically straightforward. The extended model confirms, rather than changes, their classification, leaving limited room for clinically meaningful reclassification. Therefore, the incremental value of densitometry appears greatest in the intermediate-risk range (25%–50%), where clinical uncertainty is highest and reclassification is most likely to influence management.

To facilitate clinical implementation, an online calculator was developed to allow clinicians to apply both the original and extended SUCCESS models to routine preoperative assessments (Supplementary Material).

## Discussion

This multicenter prospective study externally validated the SUCCESS score, a Scheimpflug-based model designed to predict the risk of requiring EK after cataract surgery in patients with FECD [3]. Accurate preoperative identification of patients at increased risk for postoperative corneal decompensation remains a major unmet clinical need in FECD management, as decision-making often relies heavily on slit-lamp evaluation and the surgeon’s experience [2]. The SUCCESS score addresses this limitation by providing an objective imaging-based framework that integrates the tomographic features of subclinical edema with CCT, thereby supporting individualized surgical planning.

Importantly, the endpoint of this study was the clinical indication or recommendation for EK and not its automatic performance. Some patients who meet the functional and morphological criteria may elect to defer surgery because of satisfactory binocular visual function, systemic comorbidities, or personal preference, while unexpected intraoperative endothelial trauma may lead to EK despite apparently favorable preoperative risk estimates. Therefore, the SUCCESS score should be interpreted as a likelihood estimator to support structured counseling and perioperative planning, and not as a deterministic treatment algorithm.

In this multicenter validation across six tertiary referral centers, the original model demonstrated strong discrimination, appropriate calibration, and temporal stability, confirming its reproducibility and transportability in real-world settings. Although one of the participating centers contributed to the original derivation cohort, all patients included in the present analysis were newly enrolled in a prospective multicenter design, and the original model coefficients were applied without recalibration. This fulfills the accepted criteria for external temporal and geographic validation. The incorporation of corneal densitometry provided incremental improvements in the discrimination, calibration-in-the-large, and reclassification metrics. Importantly, this translated into a correct upward reclassification of approximately one in four eyes that ultimately required EK and a correct downward reclassification of approximately one in six eyes that did not require EK—changes that may meaningfully refine risk estimation in patients near clinical decision thresholds.

### Comparison with previous studies

The tomographic variables incorporated into the SUCCESS score were derived from the morphologic classification proposed by Sun et al. at the Mayo Clinic, led by Patel, who identified three Scheimpflug-based features: loss of regular isopachs, displacement of the thinnest point, and focal posterior surface depression, as signs of subclinical corneal edema in FECD [12]. This framework enables the objective identification of early endothelial dysfunction prior to overt clinical decompensation.

In a subsequent prognostic study, the same group [13] extended this approach to estimate the 4-year risk of disease progression or surgical intervention after cataract surgery, demonstrating that risk increased with the number of tomographic abnormalities. Although both CCT (per 25-µm increment) and anterior corneal backscatter were statistically significant predictors, the authors emphasized morphological features and expressed caution regarding densitometry because of concerns regarding technical variability and inter-device standardization.

Notably, even in that model, non-standardized anterior backscatter remained an independent predictor of progression (HR ≈1.07/GSU). Although modest on a per-unit scale, this effect translates into a clinically relevant difference when comparing corneas with substantially different backscatter values; for example, a 10-unit difference (25 GSU vs. 15 GSU) would correspond to an approximate doubling of risk (HR₁₀ ≈ 1.07^10^ ≈ 1.97).

The Mayo Clinic group has established a landmark framework for tomography-based prognostication of FECD. Our study revisited this issue using a different methodological and clinical approach. In contrast to the original single-center cohort, which included a smaller sample size, a substantial proportion of bilateral eyes, and composite endpoints incorporating both clinical and subclinical progression, we focused specifically on the risk of requiring EK after cataract surgery, which is a clearly actionable clinical outcome.

Importantly, prior concerns regarding densitometry are primarily related to inter-device variability, differences in light-source intensity, calibration status, and acquisition conditions. External normalization procedures have been proposed to mitigate such variability; however, these approaches are not routinely feasible in clinical practice. In the present study, all participating centers used regularly calibrated Pentacam systems and adhered to standardized acquisition protocols under controlled and reproducible conditions. Within this harmonized framework, the densitometry values exported directly from the instrument demonstrated stable and independent predictive performance without requiring external normalization.

Peak mean densitometry within the central 3-mm zone, an operator-independent parameter available in standard Pentacam output, complemented pachymetric and posterior elevation features, resulting in measurable improvement in discrimination and reclassification.

It is important to emphasize that corneal densitometry reflects light scattered backward toward the imaging device and does not directly quantify the forward scatter reaching the retina. Therefore, the association between densitometry results and visual function should not be interpreted as causal. Rather, densitometry likely serves as a surrogate marker of stromal structural alterations, such as early edema or microstructural remodeling, which may coexist with optical changes that affect visual quality. Prior studies have reported associations between densitometry and functional parameters including visual acuity, contrast sensitivity, and higher-order aberrations [11, 14, 15]; however, these relationships reflect shared underlying pathology rather than direct optical causation.

Taken together, these findings suggest that standardized Scheimpflug imaging can yield reproducible densitometric data that provide complementary prognostic information when combined with tomographic morphology.

### Clinical implications, strengths, and limitations

The present findings highlight that objective image-based quantification can refine the preoperative risk estimation in patients with FECD undergoing cataract surgery. The SUCCESS and extended SUCCESS models translate tomographic and densitometric information into a clinically interpretable probability of postoperative EK, facilitating structured risk communication, referral planning, and surgical decision-making.

When the predicted risk is low, surgeons should proceed with standard phacoemulsification and routine follow-up. At a higher predicted risk, the model may support the consideration of combined phacoemulsification with EK or planned staged management, and help ensure that surgery is performed in centers with immediate access to corneal transplantation should decompensation occur. In intermediate-risk scenarios, where clinical uncertainty is greatest, the improved classification performance of the extended model may guide referral pathways and follow-up intensity rather than dictate a specific surgical technique. These models are intended to complement rather than replace clinical judgments.

It is important to clarify that the incremental value of densitometry does not primarily affect patients at extreme risk for whom clinical decisions are usually straightforward. Rather, its contribution is most evident among eyes classified within the intermediate-risk range, in which surgical planning is inherently uncertain. In this context, modest improvements in discrimination and reclassification may meaningfully refine risk estimation, helping clinicians to distinguish between patients who can safely undergo cataract surgery alone and those who may benefit from combined or staged management. Thus, the addition of densitometry does not aim to increase complexity for its own sake but to reduce uncertainty precisely in the subgroup where decision-making is most nuanced.

Patients with epithelial edema were excluded because this stage of corneal failure unequivocally requires keratoplasty. In contrast, all eyes with stromal edema were included regardless of clinical visibility or baseline visual acuity. This approach was intentional. First, previous studies have demonstrated that stromal corneal edema may coexist with functional vision, rendering the indications for keratoplasty less straightforward. Second, the decision to undergo EK depends not only on clinical findings but also on patient preference; excluding such cases a priori would have implied a physician-determined inevitability. Third, selective exclusion based on subjective slit-lamp assessment would have reduced reproducibility and limited applicability across centers with varying levels of corneal subspecialization.

Importantly, in some instances outside the context of the study, combined cataract surgery and EK might have been considered according to the individual surgeon’s assessments. Within the study protocol, the patients were explicitly informed about this possibility and agreed to a structured follow-up under predefined objective criteria. The short delay between cataract surgery and potential EK indications, typically weeks to a few months, did not compromise the prognosis, and donor tissue availability was ensured should transplantation become necessary. Therefore, the inclusion of these cases allowed the objective evaluation of postoperative risk under standardized conditions without adversely affecting patient outcomes.

The key strengths of this study include its prospective multicenter design, standardized imaging protocols aligned with manufacturer recommendations, and comprehensive validation strategy assessing discrimination, calibration, reclassification, and clinical utility in accordance with the TRIPOD guidelines. Although the number of EK events was modest, the cohort was larger and more heterogeneous than those in previous single-center studies [3-5,13], supporting external validity.

Limitations include restriction to tertiary centers within a single country and the use of a single Scheimpflug platform (Pentacam), which may limit the generalizability to other imaging systems or demographic settings. External validation in international and device-diverse cohorts would further clarify the transferability. Specular microscopy was performed using instruments available at each center; however, endothelial cell density was not incorporated into the predictive models, limiting the potential impact of inter-device variability on the primary analyses. As with any preoperative predictive model, intraoperative variables, such as cataract density, surgical duration, or surgeon experience, cannot be fully standardized [16,17]. However, none emerged as an independent predictor in the adjusted analyses, likely reflecting the relative homogeneity of the surgical technique and surgeon expertise across the participating centers. Therefore, predictions should be interpreted within the context of routine surgical complexity, rather than extreme surgical scenarios. Although the number of EK events was sufficient for external validation of an existing model, it remained modest, particularly for subgroup analyses and high-risk threshold decision curve estimates.

## Conclusions

In summary, this multicenter prospective study externally validated the SUCCESS score and confirmed its robustness in estimating the risk of EK after cataract surgery in patients with FECD. The addition of corneal densitometry provided incremental improvements in discrimination, calibration, and risk reclassification, particularly in clinically uncertain cases.

Both the original and extended models offer objective imaging-based tools that can be integrated into routine preoperative assessments to support structured risk estimation, referral planning, and patient counseling. By translating standardized Scheimpflug imaging parameters into a clinically interpretable probability of postoperative decompensation, the SUCCESS framework facilitates more transparent and individualized decision-making.

Further multicenter and multi-platform validation studies are warranted to assess transferability across different populations and imaging systems and to evaluate the broader clinical impact of implementation in diverse practice settings.

## Supplementary Information

Supplementary material 1.

## Data Availability

Data underlying the findings of this study are available upon reasonable request from the corresponding author and participating institutions, in accordance with institutional and ethical guidelines. An interactive, web-based calculator (SUCCESS Calculator) has been developed to facilitate clinical and research use of both the original and extended models. The calculator provides individualized estimates of the risk of requiring endothelial keratoplasty after cataract surgery in patients with Fuchs endothelial corneal dystrophy, based on the variables described in this article. Access to the calculator and its supporting documentation is provided in the Supplementary Material.

## Abbreviations

CCT: Central corneal thickness
CI: Confidence interval
DCA: Decision curve analysis
EK: Endothelial keratoplasty
FECD: Fuchs endothelial corneal dystrophy
GSU: Gray-scale unit
HR: Hazard ratio
IDI: Integrated discrimination improvement
IQR: Interquartile range
NRI: Net reclassification index
ROC: Receiver operating characteristic
SUCCESS: SUbClinical Corneal Edema Scheimpflug Study
TRIPOD: Transparent reporting of a multivariable prediction model for individual prognosis or diagnosis

## References

[1] Viberg A, Samolov B, Claesson Armitage M, Behndig A, Byström B. Incidence of corneal transplantation after phacoemulsification in patients with corneal guttata: a registry-based cohort study. J Cataract Refract Surg. 2020;46(7):961–6. 32271268 10.1097/j.jcrs.0000000000000207

[2] Sun SY, Wacker K, Baratz KH, Patel SV. Determining subclinical edema in Fuchs endothelial corneal dystrophy: revised classification using Scheimpflug tomography for preoperative assessment. Ophthalmology. 2019;126(2):195–204. 30153944 10.1016/j.ophtha.2018.07.005

[3] Arnalich-Montiel F, de-Arriba-Palomero P, Muriel A, Mingo-Botín D. A risk prediction model for endothelial keratoplasty after uncomplicated cataract surgery in Fuchs endothelial corneal dystrophy. Am J Ophthalmol. 2021;231:70–8. 33951443 10.1016/j.ajo.2021.04.016

[4] Arnalich-Montiel F, Mingo-Botín D, De Arriba-Palomero P. Preoperative risk assessment for progression to descemet membrane endothelial keratoplasty following cataract surgery in Fuchs endothelial corneal dystrophy. Am J Ophthalmol. 2019;208:76–86. 31369719 10.1016/j.ajo.2019.07.012

[5] Van Cleynenbreugel H, Remeijer L, Hillenaar T. Cataract surgery in patients with Fuchs’ endothelial corneal dystrophy: when to consider a triple procedure. Ophthalmology. 2014;121(2):445–53. 24289914 10.1016/j.ophtha.2013.09.047

[6] Kopplin LJ, Przepyszny K, Schmotzer B, Rudo K, Babineau DC, Patel SV, et al. Relationship of Fuchs endothelial corneal dystrophy severity to central corneal thickness. Arch Ophthalmol. 2012;130(4):433–9. 22491913 10.1001/archophthalmol.2011.1626 PMC3859299

[7] Collins GS, Reitsma JB, Altman DG, Moons KGM. Transparent reporting of a multivariable prediction model for individual prognosis or diagnosis (TRIPOD): The TRIPOD Statement. BMC Med. 2015;13:1. 25563062 10.1186/s12916-014-0241-z PMC4284921

[8] Barraquer RI, Pinilla Cortés L, Allende MJ, Montenegro GA, Ivankovic B, D’Antin JC, et al. Validation of the Nuclear Cataract Grading System BCN 10. Ophthalmic Res. 2017;57(4):247–51. 28288454 10.1159/000456720

[9] Cassard SD, Patrick DL, Damiano AM, Legro MW, Tielsch JM, Diener-West M, et al. Reproducibility and responsiveness of the VF-14: an index of functional impairment in patients with cataracts. Arch Ophthalmol. 1995;113(12):1508–13. 7487617 10.1001/archopht.1995.01100120038005

[10] Elhardt C, Aamoon AZ, Hartmann LM, Grün CMS, Wolf A, Wertheimer CM. Reduced quality of life in corneal dystrophy – a prospective case control study. BMC Ophthalmol. 2025;25(1):341. 40537778 10.1186/s12886-025-04200-x PMC12180281

[11] Shah K, Eghrari AO, Vanner EA, O’Brien TP, Koo EH. Scheimpflug corneal densitometry values and severity of guttae in relation to visual acuity in Fuchs endothelial corneal dystrophy. Cornea. 2022;41(6):692–8. 35175018 10.1097/ICO.0000000000002762 PMC8857507

[12] Patel SV, Winter EJ, McLaren JW, Bourne WM. Objective measurement of backscattered light from the anterior and posterior cornea in vivo. Invest Ophthalmol Vis Sci. 2007;48(1):166–72. 17197529 10.1167/iovs.06-0767

[13] Patel SV, Hodge DO, Treichel EJ, Spiegel MR, Baratz KH. Predicting the prognosis of Fuchs endothelial corneal dystrophy by using Scheimpflug tomography. Ophthalmology. 2020;127(3):315–23. 31685256 10.1016/j.ophtha.2019.09.033

[14] Kai C, Oie Y, Nishida N, Doi S, Fujimoto C, Asonuma S, et al. Associations between visual functions and severity gradings, corneal scatter, or higher-order aberrations in Fuchs endothelial corneal dystrophy. Invest Ophthalmol Vis Sci. 2024;65(6):15. 38848076 10.1167/iovs.65.6.15 PMC11166222

[15] Poh SSJ, Tey KY, Peh GSL, Neo DJH, Htoon HM, Lee EKL, et al. Assessment of early Fuchs endothelial corneal dystrophy and CTG trinucleotide expansion positivity using Scheimpflug imaging. Ophthalmol Sci. 2025;5(5):100830. 40662160 10.1016/j.xops.2025.100830 PMC12256313

[16] Perone JM, Luc MS, Zevering Y, Vermion JC, Gan G, Goetz C. Narrative review after post-hoc trial analysis of factors that predict corneal endothelial cell loss after phacoemulsification: tips for improving cataract surgery research. PLoS One. 2024;19(3):e0298795. 38512953 10.1371/journal.pone.0298795 PMC10956851

[17] O’Brien PD, Fitzpatrick P, Kilmartin DJ, Beatty S. Risk factors for endothelial cell loss after phacoemulsification surgery by a junior resident. J Cataract Refract Surg. 2004;30(4):839–43. 15093647 10.1016/S0886-3350(03)00648-5

