# Supplementary material for: Validation and extension of the SUCCESS score for Fuchs dystrophy after cataract surgery
Source: Eye Vis (Lond). 2026 Jul 10;13:30. doi: 10.1186/s40662-026-00501-4 (PMC13352704; doi:10.1186/s40662-026-00501-4)
Supplement: Supplementary file 1 — Supplementary material 1. [file 40662_2026_501_MOESM1_ESM.docx]

**Supplementary Material**

**Online SUCCESS Calculator**An interactive web-based calculator is available to apply the original and extended SUCCESS models in clinical practice:
https://arnalich.github.io/SUCCESS-Calculator/
The calculator provides predicted probabilities of requiring endothelial keratoplasty after cataract surgery based on Scheimpflug-derived parameters and corneal densitometry.

**Supplementary Figure S1. Representative Pentacam HR densitometry report used to identify peak mean corneal densitometry.** Representative Scheimpflug densitometry map showing the location of the peak mean corneal densitometry value within the central 3-mm apex-centered zone. The highlighted value corresponds to the parameter used for the extended SUCCESS model and is expressed in gray-scale units (GSU).

**
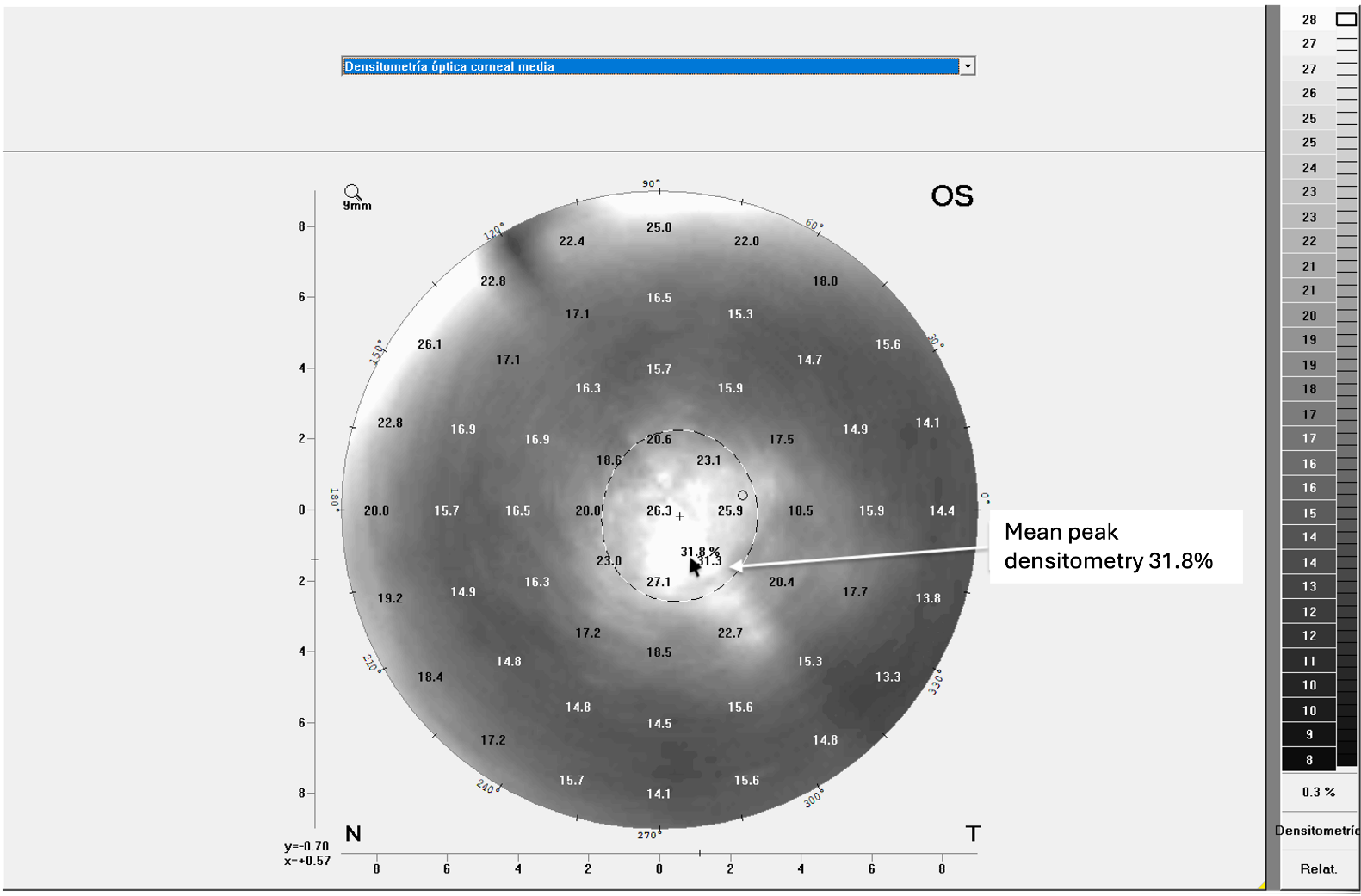
**

**Supplementary Figure S2. Receiver operating characteristic curve for peak mean corneal densitometry.** Receiver operating characteristic (ROC) curve evaluating peak mean corneal densitometry within the central 3-mm zone for predicting endothelial keratoplasty indication after cataract surgery. The optimal cutoff identified by the Youden index was 21.2 GSU, with a sensitivity of 0.79 and specificity of 0.82. The area under the curve was 0.85

**
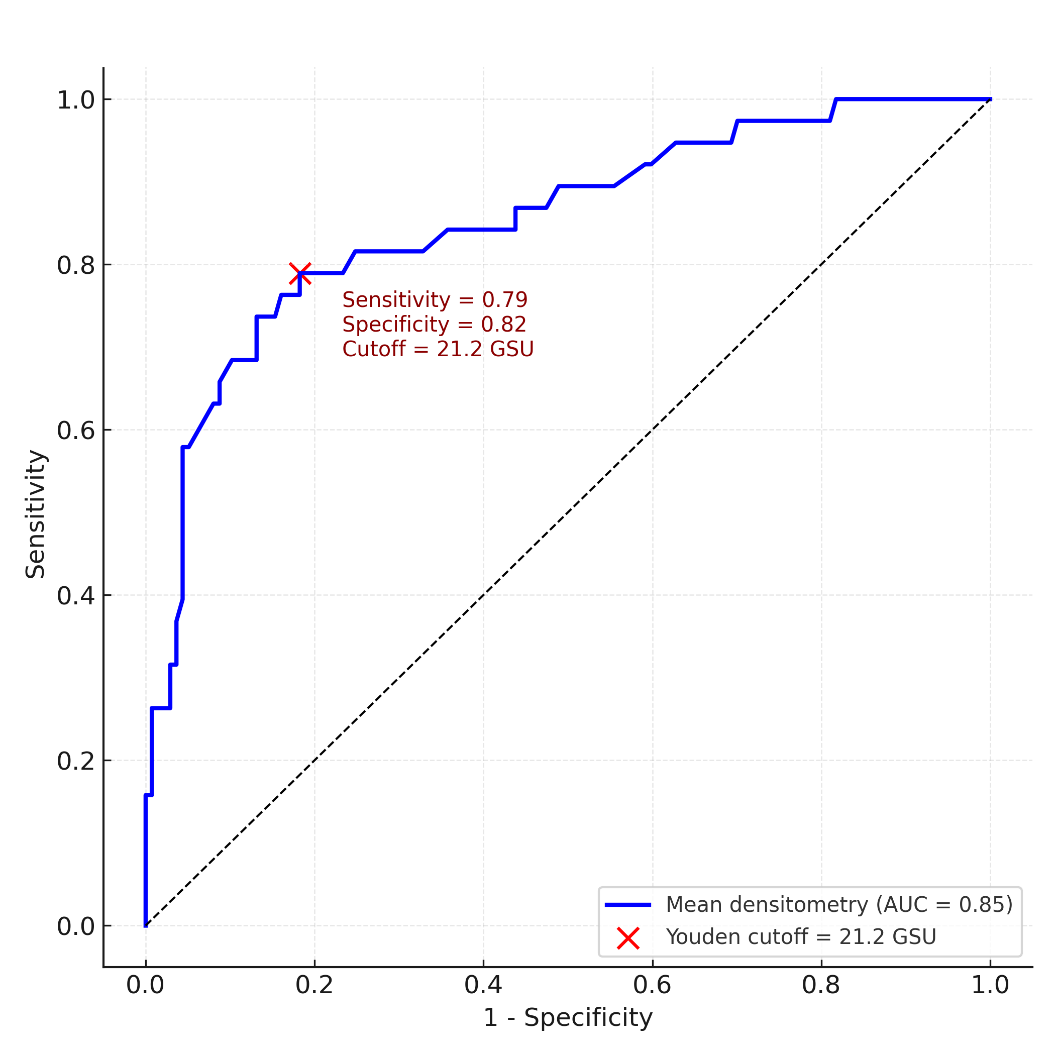
**

**Supplementary Figure S3. Evolution of the Brier score over time for the original and extended SUCCESS models.** Temporal evolution of the Brier score comparing the original SUCCESS score and the extended SUCCESS score incorporating corneal densitometry. Lower Brier scores indicate better predictive accuracy. The extended model showed lower Brier scores than the original model throughout most of the follow-up period, supporting improved overall prediction accuracy.

**
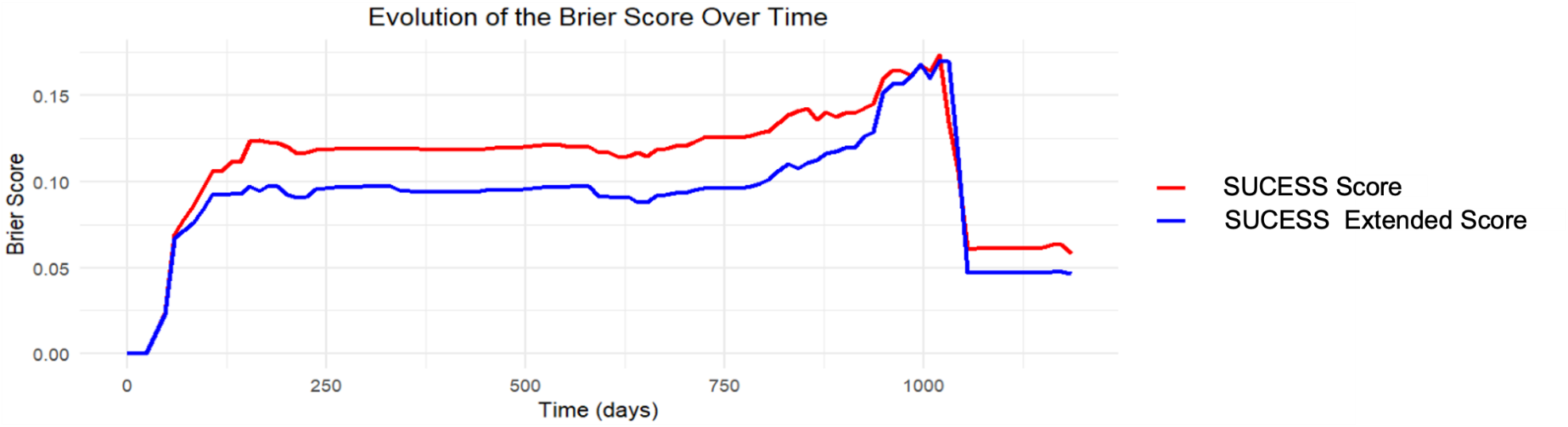
**

**Supplementary Table S1. TRIPOD summary table for the SUCCESS and extended SUCCESS models**

| TRIPOD domain | Item No. | Description for this study |
| --- | --- | --- |
| Title and abstract | 1 | Title identifies external validation + model extension. Abstract summarizes objectives, design, participants, predictors, outcomes, and main performance metrics. |
| Introduction | 2a – 2b | Clinical need in FECD is described; objectives are: (1) external multicenter validation of the SUCCESS Score and (2) evaluation of incremental value of corneal densitometry. |
| Source of data | 3a – 3b | Prospective multicenter study across six Spanish tertiary hospitals (June 2020–October 2024), with follow-up through May 2025. |
| Participants | 4a – 4c | Inclusion: adults with FECD ≥ grade 2 and visually significant cataract. Exclusion: epithelial edema requiring phaco+EK, prior corneal surgery, infection, inflammation, or comorbidities limiting follow-up. Flow diagram in Figure 1. |
| Outcome | 6a – 6b | Primary outcome: need for EK after cataract surgery, based on functional (BSCVA ≥ 0.3 logMAR or symptomatic limitation) and morphologic criteria, adjudicated by masked corneal specialists. |
| Predictors | 7a – 7b | Preoperative predictors: SUCCESS Score (tomographic edema features + CCT) and peak mean corneal densitometry (3-mm zone), categorized at >21.2 GSU. Measurements obtained under standardized Scheimpflug conditions by masked observers. |
| Sample size | 8 | 207 enrolled; 178 analyzed (38 EK events, 21%). Events-per-variable adequate for validation of a fixed-effects model with one new predictor. |
| Missing data | 9 | Case-wise exclusion. No imputation performed. Twelve percent excluded due to incomplete imaging or follow-up. |
| Statistical analysis methods | 10a – 10d | Time-to-event modeling with Cox proportional hazards. Discrimination assessed with Harrell’s C. Calibration assessed via calibration-in-the-large, slope, and Brier score at 12 months. Clinical utility assessed with decision-curve analysis. Incremental value assessed using ΔC, Brier score difference, NRI, and IDI. |
| Model development | 11a – 11b | Extended model created by adding densitometry to the fixed baseline SUCCESS Score. Coefficients estimated via Cox regression; no automated variable selection used. Predictors were evaluated individually via ROC analysis to determine optimal cut-off values. |
| Model specification | 12a – 12b | Final extended model: hazard function incorporating SUCCESS Score + dichotomized peak mean densitometry (≥21.2 GSU). Full coefficients and intercept-equivalent baseline survival at 12 months provided in Supplementary Tables. Online calculator included. |
| Model performance | 13a – 13b | Base model: C = 0.80 (95% CI: 0.73–0.86), Brier = 0.118, slope = 0.99. Extended model: C = 0.85 (95% CI: 0.79–0.90), Brier = 0.099, slope = 1.00, NRI = 0.39 (*P* = 0.002), IDI = 0.14 (*P* = 0.014). Decision-curve ΔNB = 0.057 (95 % CI: 0.006–0.113) at 50 % threshold. |
| Model validation | 14 | External validation across five geographically distinct centers. Bootstrap used only for internal estimates of extended model stability. |
| Results—participants | 15a – 15b | 178 eyes; median age 71 years; 67% women; 38 EK events. Baseline characteristics in Table 1. |
| Results—model Performance | 16 | Extended model improved discrimination, calibration, and reclassification. Calibration plots shown in Figure 3. |
| Results—clinical utility | 17 | Both models provided positive net benefit (10%–80 % thresholds); extended model superior at 25%–50 %, ≈6 additional patients correctly classified per 100. |
| Limitations | 18 | Moderate sample size; single imaging platform; Spanish tertiary setting; no international external validation yet. |
| Interpretation | 19a – 19b | SUCCESS externally validated with strong transportability. Peak mean densitometry adds predictive value without added complexity. Facilitates objective risk stratification before cataract surgery. |
| Implications and future research | 20 | International validation and integration of biomechanical or AI-derived predictors planned. |
| Supplementary information | 21 | Codebook, coefficients, calibration plot data, and online SUCCESS Calculator URL provided. |
| Funding and conflicts of interest | 22 | Institutional funding; no commercial conflicts reported. |

AI = artificial intelligence; BSCVA = best spectacle-corrected visual acuity; CI = confidence interval; CCT = central corneal thickness; EK = endothelial keratoplasty; FECD = Fuchs endothelial corneal dystrophy; GSU = gray-scale unit; IDI = integrated discrimination improvement; NRI = net reclassification index; ROC = receiver operating characteristic; SUCCESS = subclinical corneal edema Scheimpflug study; TRIPOD = transparent reporting of a multivariable prediction model for individual prognosis or diagnosis

TRIPOD summary of study design, methods, model specification, performance, and validation for the SUCCESS and extended SUCCESS models predicting the need for endothelial keratoplasty after cataract surgery in Fuchs endothelial corneal dystrophy.

**Supplementary Table S2. Diagnostic performance of densitometry parameters for predicting corneal decompensation**

| Variable (mm zone) | AUC (continuous) | AUC (categorized) | Optimal cutoff (GSU, Youden) | Odds ratio (95% CI) |  |
| --- | --- | --- | --- | --- | --- |
| Anterior (0–2 mm) | 0.81 | 0.72 | 26.4 | 6.6 (3.0–14.7) |  |
| Central (0–2 mm) | 0.79 | 0.74 | 18.2 | 8.8 (4.0–19.7) |  |
| Posterior (0–2 mm) | 0.80 | 0.73 | 16.6 | 8.5 (3.8–18.8) |  |
| Total (0–2 mm) | 0.84 | 0.77 | 21.1 | 15.6 (6.6–36.9) |  |
| Max. mean densitometry (within 3 mm) | 0.85 | 0.79 | 21.2 | 14.4 (6.1–34.3) | |

AUC = area under the receiver operating characteristic curve; CI = confidence interval; GSU = gray-scale units; Max. = maximum.

Each densitometry parameter was analyzed both as a continuous variable and as a binary variable using the Youden index to determine the optimal cutoff. The variable representing maximum mean densitometry within the 3 mm central zone showed the highest discriminative ability (AUC = 0.85).

**Supplementary Table 3. Extended baseline characteristics (variables not included in the predictive model)**

| Characteristic | All FECD patients  (n = 178) | No EK  (n = 140) | EK required  (n = 38) | *P* value |
| --- | --- | --- | --- | --- |
| 0–2 mm total densitometry (GSU), median (IQR) | 18.2 (16.0–20.8) | 17.6 (15.5–19.4) | 23.2 (20.0–26.5) | < 0.001 |
| 0–2 mm anterior densitometry (GSU), median (IQR) | 24.5 (20.0–28.4) | 23.1 (18.6–26.8) | 30.4 (25.4–37.1) | < 0.001 |
| 0–2 mm central densitometry (GSU), median (IQR) | 16.5 (14.7–18.7) | 16.0 (14.0–17.6) | 19.7 (17.5–21.0) | < 0.001 |
| 0–2 mm posterior densitometry (GSU), median (IQR) | 13.8 (11.4–16.6) | 13.4 (10.8–14.8) | 18.6 (14.2–22.3) | < 0.001 |
| Anterior chamber depth (mm), median (IQR) | 2.44 (2.12–2.79) | 2.45 (2.10–2.76) | 2.43 (2.17–2.87) | 0.37 |

EK = endothelial keratoplasty; FECD = Fuchs endothelial corneal dystrophy; GSU = gray-scale unit; IQR = interquartile range
These variables were explored but not included in the final predictive SUCCESS algorithm. Data are presented as median (IQR) unless otherwise indicated.
